# Memristive properties of hexagonal WO_3_ nanowires induced by oxygen vacancy migration

**DOI:** 10.1186/1556-276X-8-50

**Published:** 2013-01-24

**Authors:** Xiongwu He, Yanling Yin, Jie Guo, Huajun Yuan, Yuehua Peng, Yong Zhou, Ding Zhao, Kuo Hai, Weichang Zhou, Dongsheng Tang

**Affiliations:** 1 Key Laboratory of Low-dimensional Quantum Structures and Quantum Control of Ministry of Education, College of Physics and Information Science, Hunan Normal University, Changsha, 410081, People's Republic of China

**Keywords:** Electrical transport, Memristive properties, WO_3_ nanowires, Oxygen vacancies, PACS, 72.20.-i, 73.40.Qv, 73.63.-b

## Abstract

Tungsten trioxide (WO_3_) is always oxygen-deficient or non-stoichiometric under atmospheric conditions. Positively charged oxygen vacancies prefer to drift as well as electrons when the electric field is strong enough, which will alter the distribution of oxygen vacancies and then endow WO_3_ with memristive properties. In Au/WO_3_ nanowire/Au sandwich structures with two ohmic contacts, the axial distribution of oxygen vacancies and then the electrical transport properties can be more easily modulated by bias voltage. The threshold electric field for oxygen vacancy drifting in single-crystal hexagonal WO_3_ nanowire is about 10^6^ V/m, one order of magnitude less than that in its granular film. At elevated temperatures, the oxygen vacancy drifts and then the memristive effect can be enhanced remarkably. When the two metallic contacts are asymmetric, the WO_3_ nanowire devices even demonstrate good rectifying characteristic at elevated temperatures. Based on the drift of oxygen vacancies, nanoelectronic devices such as memristor, rectifier, and two-terminal resistive random access memory can be fabricated on individual WO_3_ nanowires.

## Background

Electrical switching in the electrode/oxide/electrode structure has attracted significant attention due to its rich physics and potential application in the next generation nonvolatile memory [1]. A large variety of materials (such as metal oxides, solid electrolytes, and organic materials) have been found to possess the characteristics of electrical switching [2-9]. Different models have also been proposed to understand the underlying physics of electrical switching [10-13]. However, the microscopic nature of electrical switching is still under debate, and exploring appropriate materials for fabricating two-terminal resistive random access memory (RRAM) based on electrical switching is still the most important issue. Recently, nanoscale Pt/TiO_2_/Pt switches have been fabricated and well understood by memristive switching mechanism, in which the drift of +2-charged oxygen vacancies under an applied electric field creates or annihilates conducting channels and then switches the device to on or off state [14,15]. Therefore, nonstoichiometic oxides, in which oxygen vacancies play an important role on their electronic structures, might be the most appropriate materials for fabricating next generation nanoelectronic devices.

Tungsten trioxide (WO_3_) has been investigated intensively because of its intriguing structural, electronic, and chromic properties [16-19]. Stoichiometic WO_3_ is resistive and transparent in the visible light region owing to a large band gap of 2.5 to 3.5 eV [16]. A slight deficit of oxygen (WO_3−*x*_, *x* = 1/6) is more favorable energetically than stoichiometic WO_3_ under atmospheric conditions, which implies that WO_3_ is intrinsically ‘self-doped’ by native oxygen vacancy point defects [17]. The excess electrons localized on W ions near oxygen vacancies are the major carriers, which will transport by hopping under external electric field, and then the oxygen-deficient WO_3_ will exhibit an increase in conductivity [18]. Theoretical calculations and experimental results indicate that WO_3−*x*_ films can be colored and conductive or transparent and resistive depending on the level of oxygen vacancies [16]. The memristive switching behavior in WO_3_ granular films has already been reported many times [19-22]. Single-crystalline WO_3_ one-dimensional (1D) nanostructures, with high surface-to-volume ratio and small grain size, have exhibited more outstanding electrical and chromic properties [23-25]. The drift of +2-charged oxygen vacancies in WO_3_ 1D nanostructures will influence the axial distribution of oxygen vacancies and then create or annihilate conducting channels easily, which might further enrich their electrical transport properties remarkably. Therefore, the memristive switching of WO_3_ 1D nanostructures induced by oxygen vacancies become more important not only for further understanding the physics of electrical switching but also for mass production of the RRAM devices.

In this work, we report the memristive effect induced by oxygen vacancy drift in WO_3_ nanowires with submicron length. The two-terminal Au/WO_3_ nanowire/Au devices exhibit resistive behavior under small bias voltage (electric field strength less than 10^6^ V/m) at room temperature, and memristive behavior under large bias voltage or at elevated temperature. If the two ohmic contacts between WO_3_ nanowire and two Au electrodes are asymmetric, the axial distribution of oxygen vacancies in WO_3_ nanowire can be more easily regulated with bias voltage, and then the electrical transport properties can be modulated more remarkably. The electronic devices can exhibit controllable linear resistance (up to 3 to 4 orders of magnitude) when the drift of oxygen vacancies is negligible under small bias voltage at room temperature and will exhibit asymmetric memristive effect and even good rectifying characteristic when the oxygen vacancies prefer to drift asymmetrically between two asymmetric ohmic contacts. Several nanoelectronic device prototypes, such as memristor, rectifier and two-terminal RRAM, have been proposed on individual WO_3_ nanowires.

## Methods

### Hydrothermal synthesis of WO_3_

Hexagonal WO_3_ nanowires used in this investigation, with typical diameters about 80 nm, were synthesized by aging WO_3_ sol–gel at 180°C for 48 h as previously reported [26].

### Fabrication of nanowire devices

WO_3_ nanowires were first dispersed in ethanol by sonication. Thereafter, they were deposited on a highly n-doped silicon wafer with a 100-nm SiO_2_ layer by putting one droplet of suspension on the surface. Finger electrodes were defined using a conventional photolithographic procedure and formed by evaporating 100-nm Au on the highly n-doped silicon wafer. Devices containing individual WO_3_ nanowires under at least two electrodes were selected for electrical transport measurements.

### Characterization of memristive properties

The electrical transport measurements were carried out with a Keithley SourceMeter 2602 (Keithley Instruments Inc., Cleveland, USA) on a variable temperature probe station. In order to eliminate the effect of water absorption, the probe station is placed in a homemade vacuum chamber, which can be vacuumized to a base pressure less than 10^−1^ Pa by mechanical pump, or filled with dry air or inert gases.

## Results and discussion

**Figure 1:**
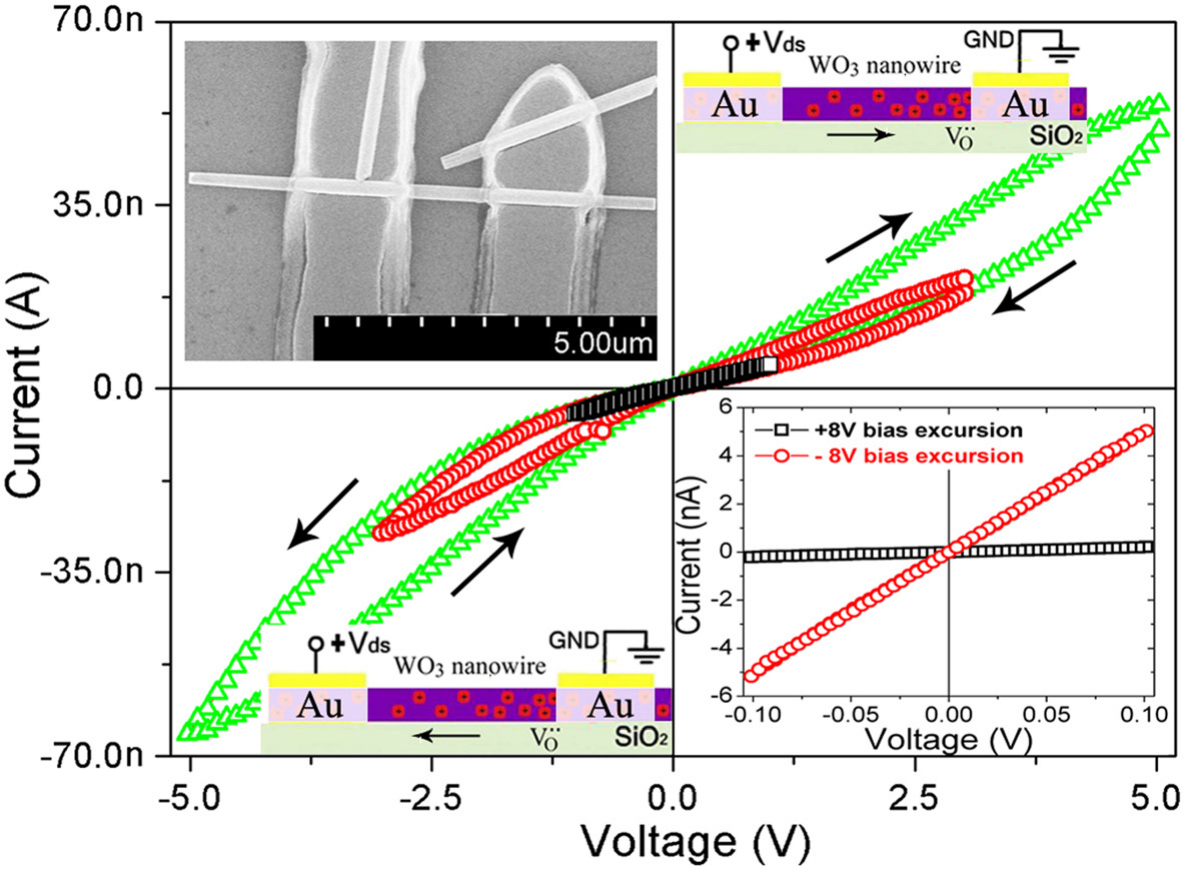
**Typical *I*-*V *curves recorded with different bias sweep ranges.** The black, red, and green curves are recorded for an individual WO_3_ nanowire at room temperature in vacuum with 1, 3, and 5 V, respectively. Inset in the upper left corner is a SEM image of the WO_3_ nanowire device. Inset in the lower right corner shows the *I*-*V* curves recorded within a small sweep range after large positive and negative bias excursion. Inset in the upper right and lower left corner are schematic diagrams showing the movement of positively charged oxygen vacancies.

Figure 1 shows typical *IV* curves recorded for an Au/WO_3_ nanowire/Au device with different bias sweep ranges in the sequence of 0→*V*_max_→0→−*V*_max_→0 at room temperature in vacuum. When the bias sweep range is small (less than 1 V), the *IV* curves is perfectly linear and symmetric, which implies that the contacts between the WO_3_ nanowire and the two Au electrodes are ohmic. At this moment, the electric field strength in the WO_3_ nanowire is about 10^6^ V/m due to the length of WO_3_ nanowire between two electrodes which is about 1 μm (upper left inset of Figure 1). As the bias sweep range increases, the *IV* curve will become nonlinear, and will not superpose itself any longer when bias voltage is swept in different directions. That is, the device is switched gradually to high resistance state under large positive bias voltage and switched back to low resistance state under negative bias voltage, which has been named as electrical hysteresis or memristive switching [14,15,27]. Figure 1 also indicates that the parts under small bias (less than 1 V) in these *IV* curves are almost linear. However, if the bias voltage is swept in the sequence of 0→−*V*_max_→0→*V*_max_→0, hysteretic-type resistive switching from the low (high) to the high (low) resistance level occurs under negative (positive) bias voltage (datum not shown here), instead of under positive (negative) bias voltage as described above. As shown in lower right inset of Figure 1, the linear resistance of the WO_3_ nanowire is about 20 MΩ, which can be switched remarkably to about 500 MΩ after being excursed under 8 V bias voltage and back to about 20 MΩ after being excursed under −8 V bias voltage. Therefore, two-terminal RRAM can be fabricated based on individual WO_3_ nanowires, which can be written by a large bias voltage and read by a small bias voltage.

**Figure 2:**
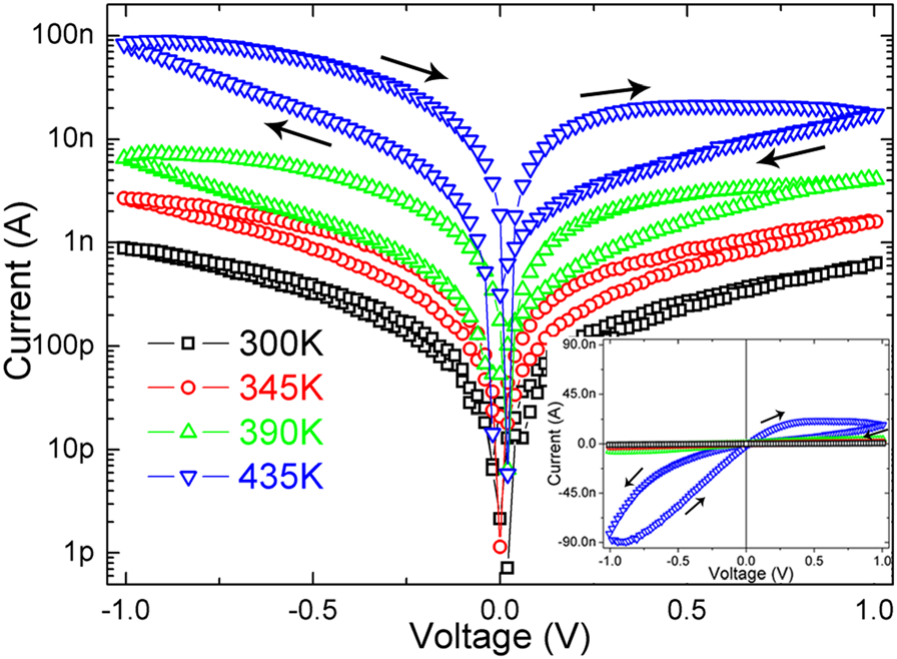
**Log-scale and linear-scale (inset) *I*-*V *curves recorded for an individual WO**_**3 **_**at different temperatures.**

Figure 2 shows the typical log-scale *IV* curves of the WO_3_ nanowire device recorded in vacuum at different temperatures ranging from 300 to 425 K. As the temperature increases, the overall resistance of the WO_3_ nanowire will decrease correspondingly, which is consistent with that of a typical semiconductor. On the other hand, the WO_3_ nanowire will exhibit hysteretic resistance switching though the bias sweep range is less than 1 V. The electrical transport properties of WO_3_ are known to be governed by the hopping conduction mechanism, and the electrons localized at the oxygen vacancies are the major carriers [1]. Theoretical calculations and experimental results indicate that the electrical transport and optical properties of WO_3−*x*_ films depend on the levels of oxygen vacancies: films with *x* > 0.2 are metallic and conductive, and those with *x* < 0.167 are transparent and resistive [17]. The oxygen vacancies act as +2-charged dopants and will drift when the electric field strength is strong enough, which will modulate the concentration distribution of oxygen vacancies and then the electrical transport properties. At room temperature, when bias voltage less than 1 V is applied to the two electrodes with a separation of 1 μm, the strongest electric field in the WO_3_ nanowire will be less than 10^6^ V/m, and the drift of oxygen vacancies is negligible. At the moment, WO_3_ nanowires exhibit resistive characteristics, and the *IV* curves are perfectly linear and symmetric. The drift of oxygen vacancies can be enhanced evidently by increasing the strength of electric field or the temperature, which will result in a change in the concentration of oxygen vacancies along the axial direction and then the resistance of the WO_3_ nanowire. The resistance of WO_3_ nanowire keeps at a minimum value when oxygen vacancies distributes uniformly along the axial direction. When the bias voltage is swept from 0 to *V*_max_ (−*V*_max_) and then back to 0, the drift of oxygen vacancies results in departure from the uniform distribution, which will lead to device switching gradually to high resistance state. When the bias voltage is swept subsequently from 0 to −*V*_max_ (*V*_max_) and then back to 0, the drift of oxygen vacancies restores the uniform distribution, which will lead to device switching gradually to low resistance state. Therefore, the critical electric field for oxygen vacancy drifting in WO_3_ nanowire is one order of magnitude less than that in its granular film [28], which might be attributed to its nanoscale diameter and single crystalline structure.

**Figure 3:**
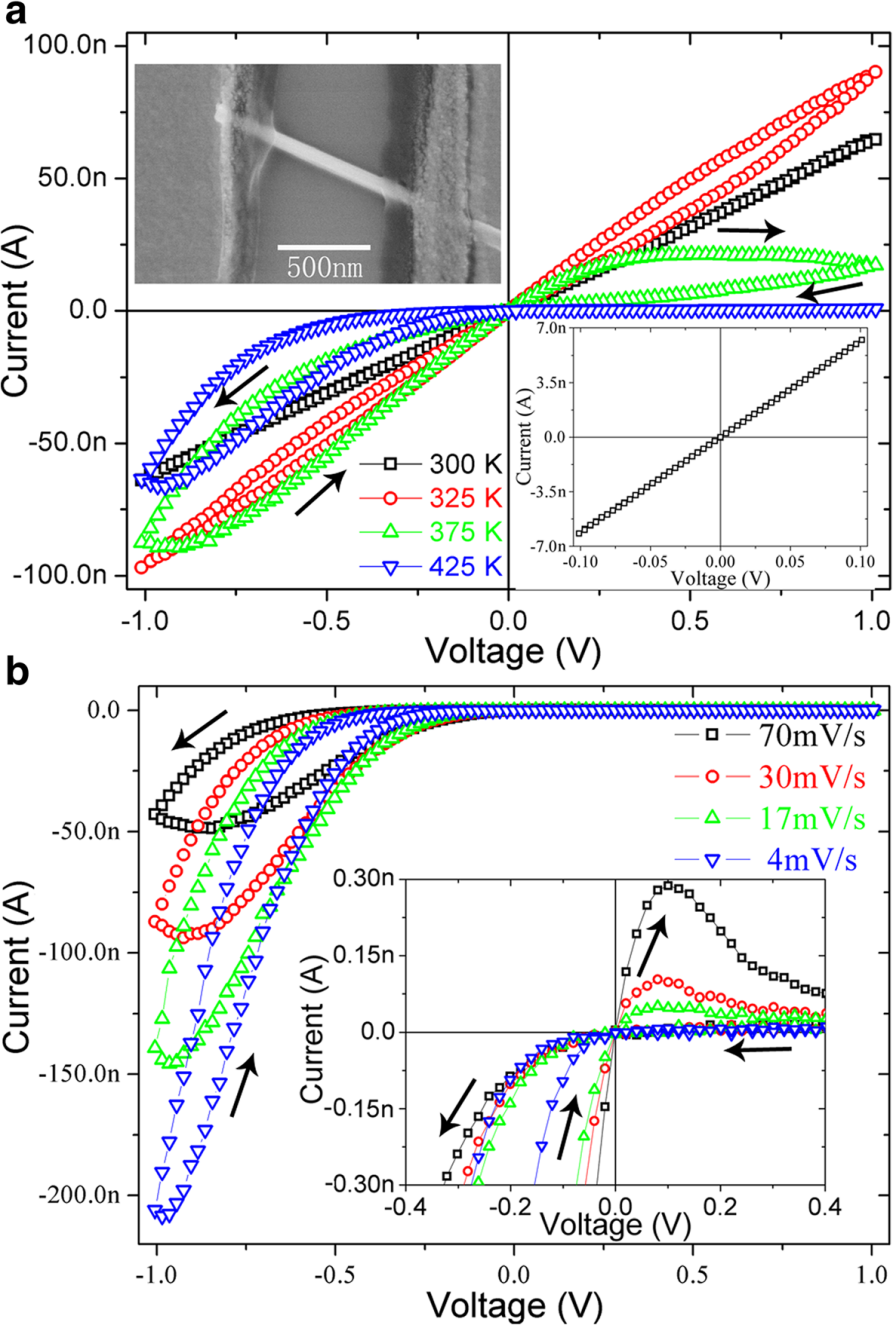
***I*-*V *curves recorded for WO**_**3 **_**nanowire with asymmetric contacts.** (**a**) *I*-*V* curves recorded for an individual WO_3_ nanowire (with a diameter of 100 nm) with asymmetric contacts between the two ends of the nanowire and electrodes under different temperatures in vacuum. Inset in the upper left corner is a SEM image of the WO_3_ nanowire with asymmetric contacts. Inset in the lower right corner shows the *I*-*V* curve recorded within a small sweep range near zero bias. (**b**) *I*-*V* curves recorded for the WO_3_ nanowire with different bias sweep rates at 425 K. Inset shows the close view of the *I*-*V* curves near zero-bias.

Another important characteristic of these *I*-*V* curves in Figure 2 is an increase in the asymmetry between positive and negative bias voltages with increasing temperature, which might be attributed to the asymmetry in the two ohmic contacts between WO_3_ nanowire and electrodes. Figure 3a shows the typical *I*-*V* curves recorded at different temperature in vacuum for the WO_3_ nanowire device with obviously asymmetric ohmic contacts. As shown in the upper left inset of Figure 3a, the asymmetric contacts mean that the segment of the WO_3_ nanowire under left electrode is very short, while the segment under right electrode is relatively long. Figure 3a indicates that the overall resistance of the WO_3_ nanowire decreases firstly, and then increases unconventionally with increasing temperature. It also indicates that these *I*-*V* curves become more nonlinear and asymmetric at elevated temperature, and the differential resistance even becomes negative in two bias ranges (near −1 and 0 V when swept from −1 to +1 V). The WO_3_ nanowire device with asymmetric contacts demonstrates good rectifying characteristic when the temperature reaches 425 K.

In order to investigate the memristive electrical switch in more detail, *I*-*V* curve was recorded at 425 K under different bias sweep rates. As shown in Figure 3b, the shape of the hysteresis loop exhibits a significant dependence on the bias sweep rate. As the sweep rate is decreased, the current will increase or decrease more quickly with bias voltage in the negative bias region, and the width of the hysteresis in bias voltage will decrease noticeably. Moreover, the current under large negative bias will increase remarkably, while the bias range with negative differential resistance (near −1 V) will also decrease correspondingly. The inset in Figure 3b shows the close view of the *I*-*V* curves near zero-bias, which indicates that the electric current increases at first, and then decreases quickly to near zero as the bias voltage is increased. It also indicates that the switch from low resistance state to high resistance state is more quickly, and the switch can be triggered by an even smaller bias voltage when the sweep rate is slowed down. These results suggest that the time scale of the memristive electrical switch might be comparable to that of bias sweep.

**Figure 4:**
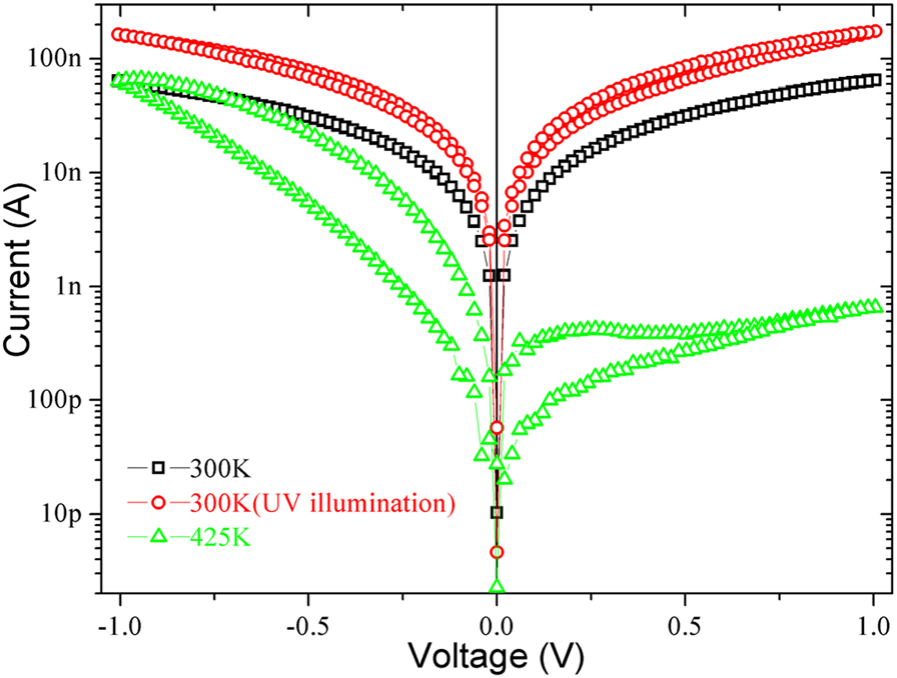
**Log-scale *I*-*V *curves recorded for comparing the effects of UV light illumination and temperature. ***I-V* curves recorded for the WO_3_ nanowire with asymmetric contacts with (circle) and without (square, triangle) UV light illumination at 300 K (square, cirle) and 425 K (triangle).

Generally, more electrons are thermally activated with increasing temperature, and the electron and hole quasi-Fermi level of the WO_3_ nanowire will rise up and lower respectively, which might alter electronic structures of the junctions between the WO_3_ nanowire and electrodes and then lead to nonlinearity and hysteresis in *I*-*V* curves discussed above. In order to clarify the effect of Fermi level shifting, UV illumination was adopted as another technique to shift Fermi level of WO_3_ nanowire by activating more electrons from valence band to conduction band. Figure 4 shows the effect of UV illumination on the electrical transport properties of WO_3_ nanowire, which indicates that the linear resistance of the nanowire decreases observably as expected, and the *I*-*V* curve remains linear, symmetric and free of hysteresis after being illuminated with 254-nm UV light. It suggests that the nonlinearity, asymmetry and hysteresis of the *I*-*V* curves have no relation with the shift of Fermi level or surface states. At elevated temperature, vibrations of the WO_3_ crystal lattice will become more violent, and the oxygen vacancies will drift more easily under external electric field as expected.

According to these results shown above, we propose a mechanism to explain the rectifying characteristic of WO_3_ nanowire devices. When the bias voltage is swept from 0 to 1 V (left electrode is positively charged) at elevated temperature, oxygen vacancies will drift toward the right electrode, and the concentration of oxygen vacancies in the segment near the left electrode will decrease rapidly because the WO_3_ nanowire segment under the left electrode is very short, which will result in a rapid increase in resistance and then a departure from linearity in *I*-*V* curve. Then, a near-stoichiometric WO_3_ nanowire segment comes into being rapidly near the left electrode and extends toward the right electrode, which will result in a remarkable decrease in electric current and negative differential resistance. When the bias voltage is swept from 1 to 0 V, the formed near-stoichiometric nanowire segment exists all the time, and the electric current dominated by electron tunnelling is very small. When the bias voltage is swept from 0 to −1 V (left electrode is negatively charged), oxygen vacancies in the nanowire near the right electrode will drift toward the left electrode, the near-stoichiometric nanowrie segment will shrink, and the concentration of oxygen vacancies in the segment near left electrode will increase continuously. The nanowire segment under the right electrode serves as oxygen vacancy reservoir, and the deposited oxygen vacancies in the reservoir have to diffuse into the nanowire segment between two electrodes firstly and then drift toward the left electrode. As a result, the current increases continuously and slowly. Therefore, the asymmetric distribution of oxygen vacancies induced by asymmetric contacts results in the asymmetric *I*-*V* characteristics. If the sweep rate is fast, for example at 70 mV/s, there is not enough time for all the deposited oxygen vacancies to diffuse out of the reservoir, which will result in a unconventional increase in the overall resistance of WO_3_ nanowire as the temperature increases (Figure 3a). If the sweep rate is very slow, for example at 4 mV/s, there is enough time for all oxygen vacancies in the reservoir to diffuse into the nanowire segment between two electrodes, which will result in a remarkable increase in the concentration of oxygen vacancies in this nanowire segment and then the conductivity. When the bias is swept from −1 to 0 V, the concentration of oxygen vacancies in the nanowire between two electrodes might increase at the very beginning all the same, and then a second bias range with negative differential resistance will come into being. As the sweep rate is slowed down, the oxygen vacancies will satuate more quickly and this bias range will shrink accordingly. Then, the concentration of the oxygen vacancies will keep constant and the nanowire exhibits linear resistance.

**Figure 5:**
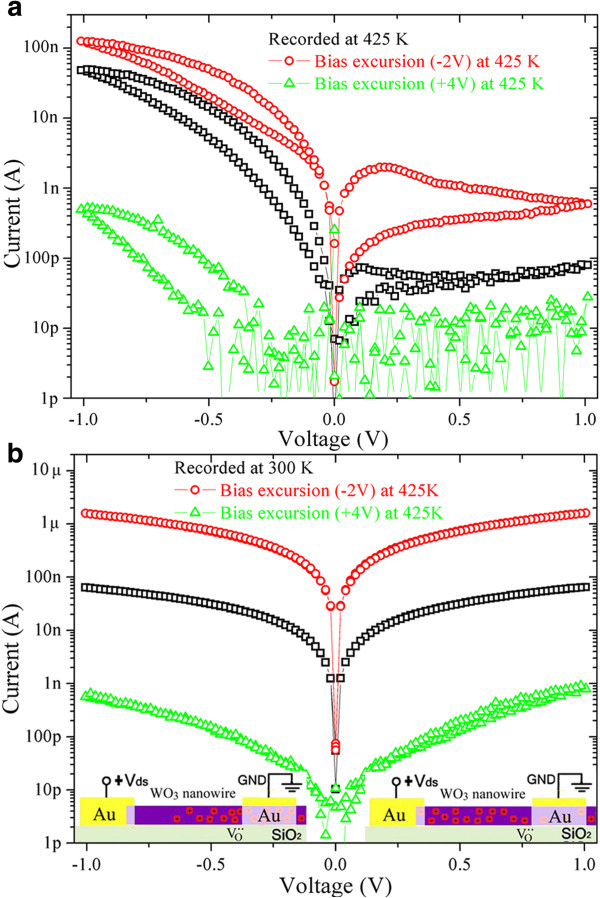
**Log-scale *I*-*V *curves recorded after being annealed at 425 K under large voltage excursions. ***I-V* curves recorded at 425 K (**a**) and at 300 K (**b**) for an individual WO_3_ nanowire with asymmetric contacts before (square) and after (circle, triangle) being annealed under large positive (+4 V) (triangle) and negative (−2 V) (cirlce) bias voltages at 425 K in vacuum. Insets at the lower left and right corner are schematic diagrams showing the distributions of positively charged oxygen vacancies.

In order to enhance the drift of oxygen vacancies, a large constant bias voltage can be applied on the device for a long time (large voltage excursions). Figure 5a indicates that the *I*-*V* curves recorded at 425 K after being annealed at 425 K under large voltage excursions remain nonlinear, nonsymmetric, and hysteretic. However, the resistance decreases overall after large negative voltage (−2 V) excursion, while it increases overall after large positive voltage (+4 V) excursion. If recorded at room temperature, the *I*-*V* curves become linear, symmetric, and free of hysteresis again (Figure 5b). However, the resistivity is about 3.39 × 10^−3^ and 16.65 Ω m obtained after large negative and positive voltage excursion, respectively (assuming that the WO_3_ nanowire has a circular cross-section). There is almost four orders of magnitude change in resistivity.

**Figure 6:**
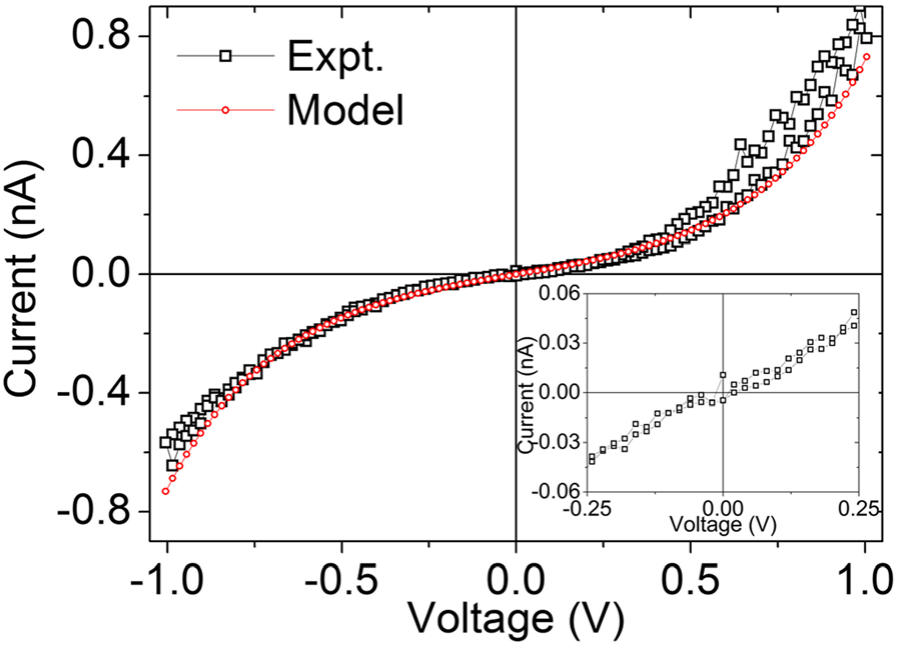
**Linear-scale *I*-*V *curve and its theoretical fitting curve recorded after being switched into high-resistance state. ***I-V* curve recorded at 300 K after being annealed under positive (+4 V) bias voltage at 425 K (the same *I*-*V* curve as that denoted by triangle in Figure 5b). Inset is an *I*-*V* curve recorded within a small sweep range near zero bias.

As shown in Figure 6, the *IV* curve denoted by triangle in Figure 5b is strictly linear only around zero bias. This *IV* curve can be well fit by an exponential function *I* ⋍ *β*sin*h*(*αV*), which is a typical characteristic of electron tunnelling (*α* and *β* are fitting constants) [15]. Therefore, a small segment of WO_3_ nanowire near one electrode might become near-stoichiometric indeed after being annealed at 425 K under positive bias voltage. This near-stoichiometric WO_3_ nanowire segment is devoid of charge carriers and then electrons can only pass through by tunneling, which results in a notable increase in resistivity of WO_3_ nanowire.

## Conclusions

In summary, the electrical transport properties of two-terminal Au/WO_3_ nanowire/Au devices depend on bias sweep range, temperature, and the symmetry of the two ohmic contacts due to the drift of oxygen vacancies under strong electric field. These devices exhibit resistive behavior under small bias voltage at room temperature and memristive behavior at elevated temperature or under large bias sweep range. If the two ohmic contacts are asymmetric, the concentration distribution of oxygen vacancies along the axial direction of WO_3_ nanowire can be more easily regulated, and then the electrical transport properties can be modulated remarkably. The electronic devices can exhibit controllable linear resistance (up to four orders of magnitude) when the drift of oxygen vacancies is negligible, and will exhibit asymmetric memristive effect and rectifying characteristic when the oxygen vacancies prefer to drift. Based on the drift of oxygen vacancies, several nanodevice prototypes (such as memristor, rectifier, and two-terminal RRAM) have been proposed on individual WO_3_ nanowires.

## Competing interests

The authors declare that they have no competing interests.

## Authors’ information

XH, YY, YP, YZ, and DZ are graduate students. JG is an undergraduate student from the Physics Department. KH and WZ are assistant professors. HY is an associate professor. DT is a professor.

## Authors’ contributions

XH and YY made the *I*-*V* measurement and drafted the manuscript. JG and HY prepared the nanowires. YP and DZ made the SEM and TEM observations. YZ, KH, and WZ fabricated the devices. DT provided the idea and completed the manuscript. All authors read and approved the final manuscript.
